# Epidemiological and clinical characteristics of congenital pseudarthrosis of the tibia in China

**DOI:** 10.3389/fped.2022.943917

**Published:** 2022-08-25

**Authors:** Yijun Zhou, Qian Tan, Kun Liu, Yaoxi Liu, Guanghui Zhu, Haibo Mei, Ge Yang

**Affiliations:** ^1^The First Affiliated Hospital of Ji’nan University (Guangzhou Overseas of Chinese Hospital), Guangzhou, China; ^2^The First People’s Hospital of Changde, Guangde Clinical Institute of Xiangya Medical College of South Central University, Changde, China; ^3^Department of Orthopedic Surgery, The Hunan Children’s Hospital, Changsha, China

**Keywords:** clinical characteristics, congenital pseudarthrosis of the tibia, epidemiological features, retrospective study, incidence

## Abstract

**Background:**

Congenital pseudarthrosis of the tibia (CPT) is a refractory and rare disease. Because of its extremely low incidence, little is known about its clinical features. In this retrospective study, we aim to analyze the clinical characteristics of patients with CPT.

**Materials and methods:**

This is a retrospective study of children with CPT identified by the radiological review. Investigations of CPT included general conditions, the characteristics of CPT, treatment methods, and surgical complications.

**Results:**

We collected 514 CPT cases from March 1999 to March 2020 in our hospital, such as 317 (61.67%) boys, 197 (38.33%) girls; 330 (62.86%) in Crawford IV; 510 (97.14%) in mid and distal 1/3 tibia; 481 (93.58%) in less than 3 years at onset age; 297 (57.78%) in less than 3 years at the first outpatient visit. The most common post-operative complication was ankle valgus (101, 39.60%), followed by limb length discrepancy (91, 35.69%), refracture (38, 14.90%), osteomyelitis (15, 5.88%), and removal of internal fixation (10, 3.93%).

**Conclusions:**

CPT with a higher incidence of Crawford IV frequently occurs in boys and the middle or distal part of the tibia; most patients have the onset age and first outpatient visit before 3 years; the major surgical complications are ankle valgus and limb length discrepancy.

## Introduction

### Background

Congenital pseudarthrosis of the tibia (CPT) is a rare disease of the skeletal system in children, with a prevalence of 1 in 140,000 or 300,000 (1, 2). CPT-related healthcare costs, work or study loss, and psychosocial problems place tremendous burdens on the families of affected children (3). As the most populous country in the world, however, little information regarding CPT epidemiology is available in China.

Due to its low incidence, clinical knowledge of CPT remains scarce and unified understanding is lacking. To this day, only three studies with more than 100 cases, two of which are multi-centered and the other single-centered, were reported (4–6). The incidence of CPT was estimated to be approximately 1 in 140,000, which was reported in a 1972 study with only 13 patients (2). The only large-sample data is a multi-center study (7) organized by The European Pediatric Orthopedic Society (EPOS) in 2000, which collected 340 cases from 20 hospitals in 13 countries. However, the above data are all from European countries, and the different findings were based on small sample sizes. Additionally, to date, there has been no population-based epidemiological study investigating of CPT. Therefore, a retrospective study based on a large sample should be conducted to better explore and enrich the relevant clinical information and knowledge of CPT.

Since the first CPT patient was admitted to our department in 1999, more than 600 patients with CPT have been treated in our unit (8–18). Therefore, based on a large number of cases, we established a Hunan CPT study database. The Hunan CPT study, which is a retrospective study of a general population sample of mainland Chinese, is designed to enhance current knowledge of CPT. The aim of the present paper is to introduce the clinical characteristics of patients with CPT.

## Materials and methods

### Study design and procedures

We performed a retrospective study on CPT cases between 1999 and 2020 in the Hunan Children’s Hospital. Patients who had CPT were identified by the radiological review. All patients visited our hospital. The studies involving human participants were reviewed and approved by the Institutional Review Board of Ethics Committee of Hunan Children’s Hospital (protocol code HCHLL-2019-37). All the patients provided written informed consent.

Investigations of CPT included the patient’s general condition (affected side, onset age, age at first outpatient visit, age at first operation and whether combined with NF1 fibular cysts, definite injury, and first visit to our hospital), the characteristics of CPT Crawford classification, the location of the pseudarthrosis, the location of the pseudarthrosis, lateral proximal tibial angle (LPTA), lateral distal tibia angle (LDTA), treatment methods, and surgical complications.

### Inclusion and exclusion criteria

We included participants aged under 16 years and provided complete data radiological review measurements. The accurate diagnosis was made by patients’ medical history, physical examination, and imaging with no trauma or birth deformity of the lower limb (19). We excluded participants aged older than 16 years or other cases caused by osteomyelitis, trauma, and malignant tumor.

### Measurements

The location of CPT was recorded as proximal 1/3, mid 1/3, and distal 1/3. Briefly, we divided the tibial length into 3 equal parts and recorded the pseudarthrosis site in each of the proximal, middle, or distal part in X-ray, named the proximal 1/3, mid 1/3, and distal 1/3. The measurement methods: at lateral X-rays, the length of the pseudarthrosis to the proximal tibial epiphyseal growth plate (a) and the length of the pseudarthrosis to the distal tibial epiphyseal growth plate (b), calculates a/(a + b) = c (0 < c < 1). C < 1/3, the pseudarthrosis was recorded as proximal 1/3 of the tibia; 1/3 ≤ C < 2/3, the pseudarthrosis was recorded as mid 1/3 of the tibia; C ≥ 2/3, the pseudarthrosis was recorded as the distal 1/3 of the tibia. The location of the pseudarthrosis of the fibular was measured in the same way. They were recorded as proximal 1/3, mid 1/3, and distal 1/3 of the fibula.

Crawford classification was recorded by anteroposterior or lateral X-rays of the tibia/fibula which were taken at the first visit. We referred to the Springer B et al. method (20) to define LDTA and LPTA. The CPT pathological classification referred to the Crawford method (21) and we defined them into 4 types.

### Statistical analysis

The general data of the corresponding children were entered into an EXCEL 2019(Microsoft^®^ Excel^®^ 2019MSO). All data were analyzed using STATA (Version 13.0, StataCorp LP, TX, United States). Two-sample *t*-tests were used to analyze continuous data, and the chi-square test was used for categorical data.

## Results

### Summary of demographic characteristics

There were no statistical differences between genders in terms of the affected side, onset age, age at first treatment, age at first operation, NF-1 (Yes\No), and first treatment in our hospital (Yes\No). Between 1999 and 2020, there were 514 children with a recorded diagnosis of CPT: 317 (61.67%) boys, 197 (38.33%) girls; 253 (49.22%) in left, 250 (48.63%) in right, and 11 (2.15%) in bilateral of affected side; 368 (71.60%) birth to <1 year, 113 (21.98%) 1 to 3 years and 33 (6.42%) over 3 years at onset age; 66 (12.84%) birth to <1 year, 231 (44.94%) 1 to 3 years and 217 (42.22%) in over 3 years at first outpatient visit; 51 (11.70%) birth to <1 year, 170 (38.99%) 1 to 3 years and 215 (49.31%) over 3 years at first operation; 349 (67.90%) with NF-1, 165 (32.10%) without NF-1; 456 (88.72%) at first treatment in our hospital, 58 (11.28%) at first treatment not in our hospital; 388 (75.49%) without definite injury, 126 (24.51%) with definite injury, which included 81 (64.29%) walking fall, 9 (7.14%) bruise with weight, 8 (6.35%) sprain, 4 (3.17%) aggravating activities, and 24 (19.05%) falling accidents (Table 1).

**TABLE 1:** Summary of demographic characteristics.

	Male (*n* = 317)	Female (*n* = 197)	Statistics	*P*-value
**Affected side**				
Left	146	107	3.5675	0.168
Right	163	87		
Bilateral	8	3		
**Onset age**				
Birth to < 1 year	235	133	3.0079	0.22
1 to 3 years	65	48		
Over 3 years	17	16		
**Age of the first visit of outpatient**				
Birth to < 1 year	38	28	1.537	0.464
1 to 3 years	149	82		
Over 3 years	130	87		
**Age of first operation**				
Birth to < 1 year	31	20	1.21	0.54
1 to 3 years	113	57		
Over 3 years	132	83		
**NF-1 (Yes/No)**				
Yes	212	137	0.396	0.529
No	105	60		
**First visit of outpatient in our hospital (Yes/No)**				
Yes	281	175	0.00612	0.9376
No	36	22		
**With/without definite injury**				
Yes	72	54	1.4493	0.229
No	245	143		
Walking fall	51	30		
Bruise with Weight	7	2		
Sprain	7	1		
Aggravating Activities	3	1		
Falling accidents	4	20		

### The radiologic features of congenital pseudarthrosis of the tibia

There were no statistical differences of Crawford classification, cystic changes of the fibula, the location of the pseudarthrosis, LPTA, and LDTA in terms of gender. But there were statistical differences of pseudarthrosis of the fibula in terms of gender (*p* = 0.015). Among the 525 limbs of CPT: Crawford type IV had the most (330), accounting for 62.86% of all types, and the other types were Crawford type I (33, 6.28%), Crawford type II (106, 20.19%), and Crawford type III (56, 10.67%). For the pseudarthrosis and fibular condition, 276 (53.70%) had CPT with pseudarthrosis of the fibula and 238 (46.30%) had CPT without pseudarthrosis of the fibula; 44 (8.56%) had CPT with cystic changes of the fibula and 470 (91.44%) had CPT without cystic changes of the fibula. Among the 525 limbs of CPT, the main locations of the lesion were in the middle and distal tibia: 15 (2.86%) in proximal 1/3 tibia, 191 (36.38%) in mid 1/3 tibia, and 319 (60.76%) in distal 1/3 tibia. Genu valgum and ankle valgus were the main manifestations of CPT (101 cases, 38.11%). Only pseudarthrosis of the fibula was statistically different between boys and girls (*p* = 0.015) (Table 2).

**TABLE 2:** The characteristics of CPT.

	Male (*n* = 317)	Female (*n* = 197)	Statistics	*P*-value
**Crawford classification**				
I	20	12 + 1	1.78	0.618
II	54 + 6	45 + 1		
III	33 + 3	18 + 2		
IV	202 + 7	119 + 2		
**Pseudarthrosis of the fibula (Yes/No)**				
Yes	183	93	5.9228	0.015*
No	134	104		
**Cystic changes of the fibula (Yes/No)**				
Yes	29	15	0.3653	0.546
No	288	182		
The location of the pseudarthrosis*				
Proximal 1/3	7	7 + 1	3.3066	0.191
Mid 1/3	110 + 2	76 + 3		
Distal 1/3	192 + 14	111 + 2		
**LPTA and LDTA**				
LPTA	86.91 ± 5.002	86.98 ± 5.456	0.1437	0.8858
LDTA	87.573 ± 10.928	87.079 ± 10.432	−0.5088	0.6111

*n*1 + *n*2 means *n*1 = unilateral and *n*2 = bilateral. *These data represents the number of affeced limbs.

### Treatment methods and surgical complications

There were no statistical differences of treatment methods or surgical complications in terms of gender. In our database, 78 (15.18%) cases received conservative treatment and 436 (84.82%) cases received surgical operation. The incidences of surgical complications were as follows: 101 (39.60%) cases occurred in the ankle valgus, 91 (35.69%) cases involved limb length discrepancy, 38 (14.90%) cases involved refracture, 15 (5.88%) cases involved osteomyelitis, and 10 (3.93%) cases involved the displacement of internal fixation (Table 3).

**TABLE 3:** Treatment methods and surgical complications.

	Male	Female	chi-square test	*P*-value
**Treatment methods#**				
Non-operation	41	37		
operation	276	160	3.227	0.072
Surgical complication*				
Ankle valgus	63	38	3.5205	0.475
Limb length discrepancy	56	35		
Refracture	18	20		
Osteomyelitis	8	7		
Removal of Internal fixation	7	3		

This was based on Chi-square tests. #degree of freedom 1, *degree of freedom 4.

### The map of regional distribution

**FIGURE 1:**
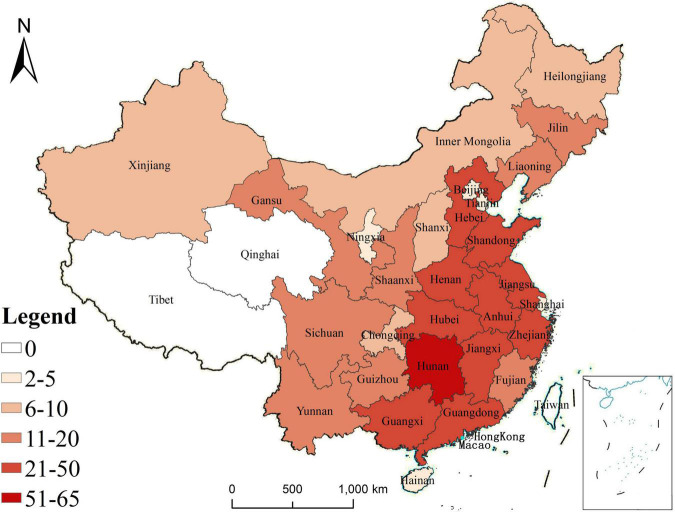
Regional distribution of CPT patients included in current analysis.

The regional distribution of patients was divided by province, autonomous region, and direct jurisdiction city. A total of 514 cases came from 29 provinces or autonomous regions or direct jurisdiction cities, such as 65 cases in Hunan province, 43 cases in Henan province, 33 cases in Shandong province, 29 cases in Guangxi Zhuang Autonomous Region, 28 cases in Jiangsu province, 27 cases in each of Jiangxi province and Hubei province, 25 cases in each of Guangdong province, Hebei province and Zhejiang province, 21 cases in Anhui province, 17 cases in each of Gansu province, Liaoning province and Yunnan province, 15 cases in shaanxi province 14 cases Fujian province, 13 cases in Jilin province, 12 cases in Sichuan province, 11 cases in Guizhou province, 8 cases in each of Shanxi province and Xinjiang Uygur Autonomous Region, 6 cases in each of Heilongjiang province, Inner Mongolia and Chongqing, 5 cases in Beijing, 4 cases in Tianjin, 3 cases in Ningxia Hui Autonomous Region, and 2 cases in each of Hainan province and Shanghai (Figure 1).

## Discussion

In this study, we present a large CPT sample retrospective study and define cases by clinical features and radiological and clinical characteristics. Thus, we bring a deeper knowledge of CPT in the Chinese context.

We collected 514 cases from 29 provinces or autonomous regions and direct jurisdiction cities. Hunan province had the highest number of cases with 65 (12.65%), in the top 10 provinces with the largest number of cases, only Guangxi Zhuang Autonomous Region 29 (5.64%) and Hubei province 27 (5.25%) were not among the top 10 provinces or autonomous regions and direct jurisdiction cities with the largest population in China. Provinces or autonomous regions and direct jurisdiction cities with larger populations had more cases, but Hunan province was not the top one with the largest population in China, which may be related to the geographical location of our hospital.

Congenital pseudarthrosis of the tibia is a rare pathology occurring in between 1/140,000 and 1/250,000 births (22–24) and is one of the most complex orthopedic situations in pediatrics. Based on the morbidity reported above, in the last 10 years, the number of newborns in China–according to official data reports–was 157.56 million and the number of CPT patients was between 630 and 1125 cases. Of the 514 cases collected in our hospital, 424 cases were collected from 2011 to 2020, and 90 cases were collected from 1999 to 2010. This means that our one center has treated nearly 40% of national CPT cases since 2011. Since the first CPT patient was admitted to our department in 1999, more than 600 patients with CPT have been treated in our unit. We have accumulated rich experience in the treatment of CPT, and have gradually been recognized by peer pediatric orthopedic surgeons over China, as well as by the majority of patients’ families. We have also made good self-media publicity so that CPT patients in China are more and more concentrated in our hospital.

We searched CPT-related clinical study from 1944 to 2021 in Web of Science and Pubmed databases, totally, 92 literatures (excluding papers of cases less than or equal to 10) were included, and the number of cases in each paper ranged from 11 to 340. There were only three multi-center studies: F Hefti et al. have 200 cases, Hitesh Shah et al. have 118 cases, and F Vigouroux has 18 cases (4). Sofield HA is the only one with more than 100 cases in the single-center study (6). The epidemiological data compared with other studies show in Supplementary Table 1.

Currently, the timing of surgery for CPT is a controversial issue (25). EPOS recommends avoiding surgery for CPT in patients younger than 3 years old, and that operation should be postponed to the age of 5 years, otherwise, patients may have a worse prognosis (7). Harding similarly recommended deferring surgery to the age of 4 years (26). However, Shah H reported that the achievement of the bone union in young children can minimize the abnormal growth and lower limb shortening (27). Joseph reported that bone union of CPT occurred in 12 out of 13 (92%) children treated before the age of 3 years (28). Liu reported on 42 patients with CPT and the frequency of bone union was higher in children with CPT operated on before reaching 3 years of age, and suggested that there is no need to defer surgery for CPT until the child is older than 3 years of age (14). In our study, we reported 514 cases of CPT, surgery was performed in 436 patients which included 221 (50.69%) younger than 3 years old, and the patients had a good prognosis (Tables 1, 3). Considering this controversial issue, in our study, onset age, first outpatient visit and first operation were divided into three groups which were birth to 1 year, 1 to 3 years, and over 3 years. In this study, onset age before 3 years had 481 (93.58%) cases and after 3 years had 33 (6.42%) cases; the age at the first outpatient visit before 3 years had 297 (57.78%) cases and after 3 years had 217 (42.22%) cases; this phenomenon of operations before 3 years being as common as operations after 3 years may be related to the age at the first outpatient visit.

Pseudarthrosis of the fibula (CPF) is frequently associated with CPT, but it becomes uncommon when it is isolated. Isolated CPF is usually considered a less severe condition than CPT. However, its site–most frequently near the ankle–leads to severe valgus and instability of this weight-bearing joint (25–29). In Liu’s study (10), patients with fibular pseudarthrosis had a high incidence of refracture and ankle valgus, and he suggested that attention should be paid to the presence of fibular pseudarthrosis when managing CPT. There were 276 (53.70%) cases of CPF of the 514 cases in this study, and 44 (8.56%) cases with cystic changes in his fibula. There were statistical differences in the prevalence of CPF, but no statistical differences in cystic changes of the fibula.

We found that Crawford IV (330, 62.86%) CPT had the most cases, which may be associated with hyperactivity. We treated Crawford IV CPT and developed into Crawford IV CPT by operation. It has been well documented that external fixation in children and adolescents has a significant physical and physiological impact, with studies reporting pain and consequent sleeping problems in approximately half of the patients (30). The operation complications–related primarily to the use of an external or internal device, residual limb-length discrepancy, and valgus deformity–are commonly reported, with an overall complication rate of 30%–100% (31). In our study, the complications also included ankle valgus, limb length discrepancy, refracture, osteomyelitis, and removal of internal fixation. The present study is a retrospective review limited by the heterogeneity of the available data and follow-up. Firstly, this study was a cross-sectional analysis and did not provide prognostic or therapeutic recommendations for cohort studies. Secondly, this study was a single-center analysis. Although our cases came from all over the country, there was still bias. We should combine the China Pediatric Orthopedic Association with expanding multi-center research in future studies; with further detailed documentation, it may be possible to clarify many more issues.

## Conclusion

Until now, we have collected 514 cases of CPT, which constitutes the largest single-center study. CPT with a higher incidence of Crawford IV frequently occurs in boys and middle or distal tibia; the major surgical complications are ankle valgus and limb length discrepancy. In subsequent studies, we will further report pathologic mechanism, surgical methods, complications, and prognosis of CPT through prospective studies.

## Data availability statement

The original contributions presented in the study are included in the article/Supplementary material, further inquiries can be directed to the corresponding authors.

## Ethics statement

The studies involving human participants were reviewed and approved by the Institutional Review Board of Ethics Committee of Hunan Children’s Hospital (protocol code HCHLL-2019-37). All the patients provided written informed consent.

## Author contributions

YZ, GY, and HM: conceptualization and writing – review and editing. YZ, YL, and QT: methodology. KL, GZ, and YL: validation. QT and YZ: formal analysis and investigation. GY and HM: resources, project administration, and funding acquisition. YZ: data curation and writing – original draft preparation. HM and GZ: supervision. All authors have read and agreed to the published version of the manuscript.
