# Supplementary material for: Epidemiological and clinical characteristics of congenital pseudarthrosis of the tibia in China
Source: Front Pediatr. 2022 Aug 25;10:943917. doi: 10.3389/fped.2022.943917 (PMC9452726; doi:10.3389/fped.2022.943917)
Supplement: Supplementary file 1 [file Table_1.doc]

Supplement table 1 The top 20 papers of CPT from 1944 to 2021 which have

| Number | Article | Published year | Pop(n) | Mean age of follow up (year) | Operation（n） | Refracture rate(%) | Primary union rate(%) |
| --- | --- | --- | --- | --- | --- | --- | --- |
| 1 | [F Hefti et al [ PMID: 10647103]](https://pubmed.ncbi.nlm.nih.gov/?term=Hefti+F&cauthor_id=10647103) | 2000 | 340 | 14.5 | 194 | - | 75.5 |
| 2 | [Hitesh Shah et al [PMID: 29664876]](https://pubmed.ncbi.nlm.nih.gov/?term=Shah+H&cauthor_id=29664876) | 2018 | 119 | 18.2 | 119 | 33.6 | 85.7 |
| 3 | [Isao Ohnishi et al [PMID: 15718906]](https://pubmed.ncbi.nlm.nih.gov/?term=Ohnishi+I&cauthor_id=15718906) | 2005 | 73 | 11.8 | 73 | 13.7 | 73.9 |
| 4 | [Andrea Laufer et al [PMID: 33371504]](https://pubmed.ncbi.nlm.nih.gov/?term=Laufer+A&cauthor_id=33371504) | 2020 | 69 | 6.9 | 26 | 15.4 | 69.2 |
| 5 | [Guang-Hui Zhu et al [PMID: 27770774]](https://pubmed.ncbi.nlm.nih.gov/?term=Zhu+GH&cauthor_id=27770774) | 2016 | 56 | 5.2 | 56 | 26.0 | 89.2 |
| 6 | [An Yan et al [PMID: 29310362]](https://pubmed.ncbi.nlm.nih.gov/?term=Yan+A&cauthor_id=29310362) | 2017 | 51 | 1.6 | 51 | 0 | 92.2 |
| 7 | [IC Tuncay et al [PMID: 7962500]](https://pubmed.ncbi.nlm.nih.gov/?term=Tuncay+IC&cauthor_id=7962500) | 1994 | 43 | 4.8 | - | - | - |
| 8 | Anderson KS et al [PMID: 4463693] | 1974 | 40 | - | 36 | 25.0 | 52.8 |
| 9 | [Tarek A El-Gammal et al [PMID: 33448723]](https://pubmed.ncbi.nlm.nih.gov/?term=El-Gammal+TA&cauthor_id=33448723) | 2021 | 39 | 8.1 | 39 | 0 | 96.0 |
| 10 | [Claire E Shannon et al [PMID: 34202921]](https://pubmed.ncbi.nlm.nih.gov/?term=Shannon+CE&cauthor_id=34202921) | 2021 | 39 | 7.0 | 39 | 0 | 100.0 |
| 11 | [Brian T Carney et al [PMID: 12180613]](https://pubmed.ncbi.nlm.nih.gov/?term=Carney+BT&cauthor_id=12180613) | 2002 | 37 | - | 21 | - | 48.0 |
| 12 | [JA Traub et al [PMID: 10573341]](https://pubmed.ncbi.nlm.nih.gov/?term=Traub+JA&cauthor_id=10573341) | 1999 | 36 | - | 29 | - | 51.7 |
| 13 | [Huajun Deng et al [PMID: 34211603]](https://pubmed.ncbi.nlm.nih.gov/?term=Deng+H&cauthor_id=34211603) | 2021 | 36 | 6.6 | 36 | 2.8 | 97.2 |
| 14 | Nguyen NH [PMID: 19242380] | 2009 | 29 | 6.3 | 29 | 58.6 | 52.2 |
| 15 | Zumiotti A et al [PMID: 8133767] | 1994 | 27 | - | 27 | 18.5 | 85.2 |
| 16 | Joseph B [PMID: 14581777] | 2003 | 26 | - | 26 | 7.7 | 77.8 |
| 17 | Cho TJ et al [PMID: 18378925] | 2008 | 23 | - | 23 | 52.2 | 47.8 |
| 18 | Matthew BD [PMID: 15743845] | 2005 | 21 | 17.2 | 21 | 57.1 | 85.7 |
| 19 | Ahmed MT et al [PMID: 18953621] | 2008 | 20 | 4.3 | 20 | 40.0 | 100.0 |
| 20 | Sakti PD [PMID: 24827969] | 2014 | 20 | 5.0 | 20 | 35.0 | 65.0 |

detailed epidemiological data
